# Eco-Friendly Management of *Acanthoscelides obtectus* Through Individual and Combined Applications of 1,8-Cineole and Diatomaceous Earth

**DOI:** 10.3390/insects17020132

**Published:** 2026-01-23

**Authors:** Evrim Sönmez

**Affiliations:** Science Teaching, Department of Mathematics and Science Education, Faculty of Education, Sinop University, Sinop 57000, Türkiye; esonmez@sinop.edu.tr

**Keywords:** environmental friendly control, eucalyptol, binary mixtures, pest, essential oils, mortality, inhibition rate, reproductive performance

## Abstract

With growing concerns about chemical insecticides, there is also growing interest in safer, natural alternatives. This study investigated the individual and combined effects of two promising eco-friendly alternatives, diatomaceous earth (DE) and 1,8-cineole, a plant-based essential oil component, on adults of *Acanthoscelides obtectus* under laboratory conditions. Against the maximum doses of both products (DE 800 ppm, 1,8-cineole 5 ppm), *A. obtectus* showed 100% mortality within the first 24 h. Although mortality at lower doses (DE 25, 50, 100 ppm, 1,8-cineole 0.600, 1.2 ppm) began from the first day, it was not as effective. In the combined application of 200 ppm DE and 2.5 ppm 1,8-cineole, the mortality rate was 73.33 ± 8.81%, representing the most effective outcome achieved with the application of relatively low doses of DE and 1,8-cineole. No F_1_ progeny was observed at any dose of DE or in the combined applications. In contrast, F_1_ progeny occurred only at three doses of 1,8-cineole (0.150, 0.300, and 0.600 ppm); however, the number of eggs and emerging adults was markedly lower than in the control group and showed a statistically significant difference. Overall, the results obtained from DE alone and DE + 1,8-cineole applications were more effective.

## Simple Summary

Stored-product insect pests are responsible for a substantial proportion of losses in stored agricultural commodities. Increasing awareness of the adverse effects of synthetic pesticides on environmental and human health has prompted researchers to seek alternative control strategies, with particular focus on diatomaceous earth and plant-derived essential oils. In this study, the control efficacy of diatomaceous earth (DE), 1,8-cineole, and their combination was evaluated against *Acanthoscelides obtectus*, an important storage pest that causes significant damage to stored beans. Although the insecticidal properties of 1,8-cineole have been documented, its high volatility and toxicity at elevated doses restrict its practical use. To minimize these disadvantages while enhancing its efficacy, the present study explored the use of reduced doses of DE and 1,8-cineole, both individually and in combination, as a control approach for this pest. The findings demonstrate that both DE and 1,8-cineole are effective against *A. obtectus*, and that their combined application results in high mortality rates along with suppression of F_1_ progeny. Overall, the results highlight the potential for developing specialized biological formulations or application strategies that capitalize on these observed interactions to improve the protection of stored products.

## 1. Introduction

*Acanthoscelides obtectus* Say (Coleoptera: Bruchidae) is one of the most common stored product pests, especially damaging grains such as beans, chickpeas and black-eyed peas [1]. The primary host of *A. obtectus* is the common bean (*Phaseolus vulgaris*, Fabaceae) [2,3,4,5,6] and produces multiple generations per year [7]. In *A. obtectus*, the optimal temperatures are 27–29 °C for adults, 24–27 °C for larvae, and 22–26 °C for pupae. Egg development may last 30–45 days before the emergence of first-instar larvae. Larvae molt to the second instar after approximately 3 days and begin feeding on the seed; the larval period lasts a total of 3–3.5 weeks. The pupal stage occurs within the seed and is completed within 9–29 days. A single generation of the species completes its development from egg to adult within 100–110 days, and although adult longevity varies with temperature and humidity, it is generally 7–14 days; under warm storage conditions, successive generations may occur [7,8,9]. Female adults lay up to 200 eggs on bean seeds, either individually or in clusters of 4–20 [10]. The larvae that hatch from these eggs feed within the bean seeds, creating cavities that reduce the nutritional value of the grains, while also contaminating them with feces and larval body residues. This situation results in numerous adverse effects on stored grains, including weight loss, reduced germination capacity, decreased nutritional value in terms of protein content, and a decline in both product quality and market value [11,12]. In addition to insect infestations, grain products are also exposed to various other factors during storage, such as microorganisms, mites, and rodents. To reduce the extent of damage caused by these harmful organisms, producers often prefer chemical control methods due to their ease of application, rapid effectiveness, and relatively low cost [13]. It is well documented that insects have developed resistance to many active ingredients used to protect stored products, such as malathion, chlorpyrifos-methyl, fenitrothion, phosphine, pirimiphos-methyl, etrimfos, and others [14,15]. To prevent these qualitative and quantitative losses in both the nutritional and market value of grains, which hold an important place in human nutrition, and to mitigate the harmful effects of insecticides on the environment, it is essential to maintain proper storage conditions for grains during storage [14,15,16,17]. Due to these adverse effects on human and environmental health, as well as public perception, the demand for natural products with no side effects has been steadily increasing [18,19,20,21].

In addition to numerous studies on the use of natural enemies of insects, plant essential oils (EOs), and inert dusts in the management of stored-product pests [11,22,23,24,25,26,27,28,29], recent years have seen increasing attention toward control methods employing phytochemicals such as diatomaceous earth and EOs [30,31,32]. The use of DE as an insecticide dates back to as early as 2000 BC in China [33]. Diatoms are microscopic, photosynthetic algae that, after death, settle to the bottom of aquatic environments and accumulate in successive layers. These resulting sediments are called diatomaceous earth (DE) [34]. Processed DE a soft and chalk-like powder, is widely used as an insecticide due to its highly absorbent and abrasive nature. As an insecticide DE removes the waxy coating of the insect epicuticle, which serves as the primary barrier against water loss. It absorbs lipids from the cuticle and abrades its surface, leading to increased water evaporation, desiccation, and eventually death of the pest [33,35,36,37]. In addition, DE particles adhering to insect bodies have been reported to restrict movement, block the trachea, spiracles, digestive system, and genital openings, thereby reducing courtship behavior, mating, and offspring production, while also impairing basic functions such as vision and signaling [38,39]. Due to its strong insecticidal activity, long-lasting effectiveness, and low mammalian toxicity, DE has attracted considerable attention in recent years.

Although DE is considered safe and environmentally friendly, it can leave residues on stored products, cause corrosion of machinery and equipment, create difficulties in achieving uniform distribution, and may also lead to a reduction in moisture content of bean seeds [40,41]. In particular, DE can form an effective insecticidal combination when used together with the active components of EOs, which often exhibit attractant effects on insects [42,43,44,45]. Due to their rapid degradability, low impact on the environment and non-target organisms, and plant-based origin, many researchers have also focused on investigating the effects of plant EOs on harmful insects [46,47].

Secondary metabolites derived from plants have been proposed as a safer, more effective, and more sustainable alternative to traditional pest management approaches. Numerous phytochemicals have been reported to exhibit bioactivity against insects; among these, monoterpenoids, major constituents of EOs found in many aromatic plants, are part of plants’ natural chemical defense against pathogens, insects, and other herbivores [48,49]. 1,8-cineole (also known as eucalyptol) is a monoterpene oxide commonly found in aromatic plants and is characterized by its distinctive eucalyptus scent. Members of the families Lamiaceae, Myrtaceae, and Lauraceae generally contain high amounts of 1,8-cineole [50,51,52,53]. Numerous studies have demonstrated the insecticidal, repellent, antifeedant, and growth-inhibiting effects of essential oils rich in 1,8-cineole, confirming their efficacy [54,55,56,57,58]. The mechanism of action of 1,8-cineole typically involves acetylcholinesterase (AChE) inhibition and interaction with gamma-aminobutyric acid (GABA) receptors, leading to respiratory suppression via absorption through the tracheal system, muscle spasms, paralysis, and death [54,55]. In addition, it can affect gustatory and olfactory sensilla, resulting in repellent activity, growth retardation, or the development of deformed individuals. However, the use of EOs and monoterpenes is limited due to their high volatility, low water solubility, and chemical instability [59,60].

Although the insecticidal effects of diatomaceous earth (DE) on *A. obtectus* have been well documented in the literature, no studies have been found investigating the use of DE in combination with essential oil components, particularly 1,8-cineole. However, DE and 1,8-cineole possess distinct and complementary modes of action. DE disrupts the integrity of the insect cuticle, leading to water loss and physical stress, whereas 1,8-cineole is a volatile monoterpene that affects the nervous system and induces rapid toxic and behavioral responses. These different mechanisms suggest a potential for complementary insecticidal effects when the two substances are applied together. Moreover, the abrasive nature and residue formation of DE at high doses on legume seeds, as well as the strong irritant effects of 1,8-cineole at high concentrations, limit the practicality and sustainability of their sole use at elevated doses. In this context, the combined application of DE and 1,8-cineole at reduced doses is expected to maintain insecticidal efficacy while minimizing adverse effects, thereby offering a more sustainable and environmentally friendly management strategy against stored-product pests. In this study the lethal and physiological effects of DE and 1,8-cineole, both individually and in combination, on *A. obtectus* were determined. This study particularly aims to reduce the quantities of insecticidal agents used in single-application settings and to propose sustainable management strategies for insects that damage stored products [39].

## 2. Materials and Methods

### 2.1. Insect Rearing

The *A. obtectus* adults used in this study were obtained from cultures reared since 2017 at the Research Laboratory of the Faculty of Education, Sinop University. The experiments were initiated by establishing stock cultures of the bean weevil. Laboratory cultures may develop behavioral adaptations as a result of long-term rearing under controlled conditions. To minimize this effect, the cultures were periodically refreshed each year using naturally infested bean seeds obtained from local markets. Prior to the present experiment, the stock cultures were re-established approximately three months earlier using infested beans collected from a local market. Therefore, the insects used in the bioassays represented the second laboratory generation derived from field populations. Adult *A. obtectus* obtained from the main cultures were transferred into 1 L glass jars containing 500 g of Dermason-type bean seeds (*Phaseolus vulgaris*). Randomly selected insects emerging from these cultures were used in the experiments. Since the adult longevity of the bean weevil varies between 7 and 14 days depending on temperature and humidity conditions, two-day-old adults were used in this study to ensure reliable data. In addition, unsexed adults were employed to avoid possible differences between sexes. Unsexed adults refers to the use of adult insects without prior sex determination; thus, both male and female individuals were included together in each experimental unit. This approach was adopted to reflect natural population structure and to avoid potential bias related to sex-specific responses. All experiments were conducted under laboratory conditions at 25 ± 2 °C and 65 ± 5% relative humidity.

### 2.2. Diatomaceous Earth and 1,8-cineole

In the experiments, a natural DE product, Detech^®^ (Entoteam R&D Food Agriculture Industry Trade Ltd., Istanbul, Turkey), obtained from mines in the Central Anatolia Region of Türkiye (Kahramanmaraş), was used. Detech^®^ is a light gray powder composed of 80.6% SiO_2_, 4.75% CaO, 4.70% Al_2_O_3_, 1.50% Fe_2_O_3_, 0.85% MgO, 0.50% K_2_O, 0.40% Na_2_O, and <0.01% TiO_2_ [61]. The average particle size of Detech^®^ is 14.061 μm [62]. 1,8-cineole (98% purity, CAS Number: 470-82-6) was purchased from Sigma-Aldrich Chemical Company (Munich, Germany).

### 2.3. Bioassays

#### 2.3.1. Individual Application of Diatomaceous Earth

In the diatomaceous earth applications, 100 g of beans were placed in 1-L plastic bags and Detech^®^ (hereinafter referred to as Detech, diatomaceous earth, or DE) was applied directly at the specified doses. The plastic bags were tightly sealed and shaken manually for five minutes to ensure homogeneous distribution [63]. Subsequently, 20 beans were randomly selected from the treated 100 g batch, weighed (total mass per dish), and placed into a Petri dish. Then, 10 adult insects were introduced into the Petri dish. Each Petri dish represented one experimental replicate per concentration. To determine the individual effects of DE on insect mortality, DE was applied at concentrations of 25, 50, 100, 200, 400, and 800 ppm (mg DE kg^−1^ beans, *w*/*w*). Trials were set up with three replicates per treatment and repeated twice independently. Each Petri dish contained 20 bean seeds treated with the designated doses of DE and 10 adult *A. obtectus* individuals.

#### 2.3.2. Individual Application of 1,8-cineole

1,8-cineol (purity: 98%, Sigma-Aldrich Company, Germany) was prepared in ethanol at concentrations of 0.150, 0.300, 0.600, 1.2, 2.5, and 5 ppm (mg L^−1^ in ethanol). For each application, a fixed volume of the prepared solutions (1 mL per 100 g of beans) was sprayed onto 100 g of beans, ensuring uniform coverage of the bean surface. After waiting 1 min for the solvent to evaporate, 20 beans were randomly selected from each treatment, weighed and placed into a single Petri dish, with each Petri dish considered one experimental replicate. Then, 10 adult *A. obtectus* individuals were introduced into each Petri dish. In this study, the reported ppm values refer to the concentrations of 1,8-cineole solutions prepared in ethanol. The trials were set up with three replicates per concentration, and all treatments were repeated twice independently.

#### 2.3.3. Combined Applications of DE + 1,8-cineole

For the combination treatments, 1,8-cineole was first applied to the beans, followed by DE, as described above. Trials were set up with three replicates per treatment, and the treatments were repeated two times. Each Petri dish contained 20 bean seeds treated with the designated doses of DE + 1,8-cineole and 10 adult *A. obtectus* individuals.

To determine the combined effect of DE and 1,8-cineole on insect mortality, beans were treated with the following mixtures:

25 ppm DE + 0.600 ppm 1,8-cineole
25 ppm DE + 2.5 ppm 1,8-cineole
200 ppm DE + 0.600 ppm 1,8-cineole
200 ppm DE + 2.5 ppm 1,8-cineole

The Petri dishes were examined on days 1, 2, 3, 4, 7, and 14 after treatment, and dead insects were recorded to determine mortality rates. Insect mortality was determined by gently prodding the insects with a fine hair brush; individuals showing no movement were considered dead. In the control groups, no treatment was applied.

### 2.4. Effect on F_*1*_ Progeny

**Figure 1:**
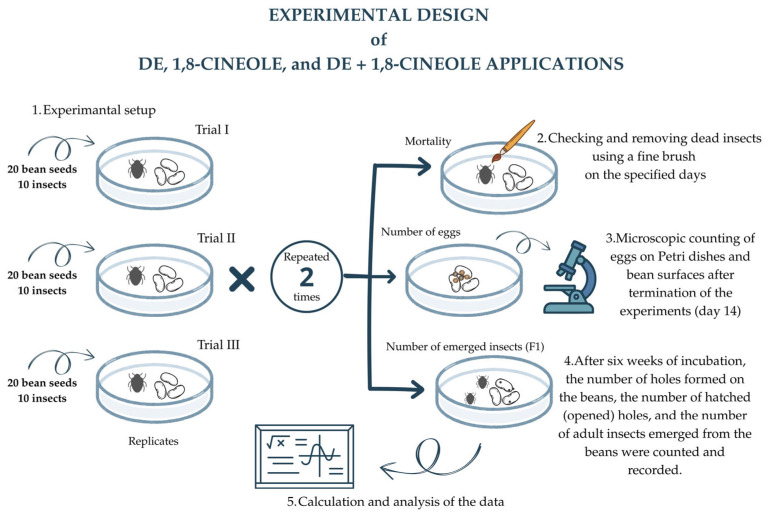
Overview of the experimental workflow applied uniformly to each treatment type (DE, 1,8-cineole, and DE + 1,8-cineole) and to all concentration levels tested against *Acanthoscelides obtectus*. The figure illustrates the replication scheme, mortality assessments, post-treatment egg counts, and subsequent evaluation of bean damage and F_1_ progeny after incubation.

**Figure 2:**
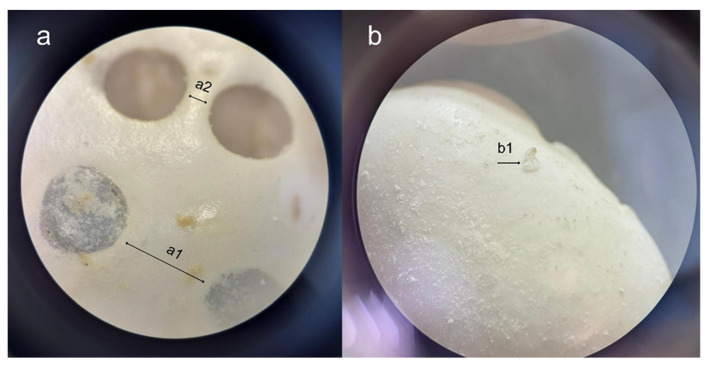
Examples of eggs and holes recorded at the end of the experiments.

After 14 days, the mortality trials were terminated. Following the completion of mortality assessments, all insects (both live and dead) were removed from the Petri dishes, and the eggs present on the bean surfaces and Petri dishes were counted under a stereomicroscope (Leica Z4, Bend Engineering Medical Devices Industry and Trade Ltd., Samsun, Türkiye). Subsequently, the beans and Petri dish surfaces were incubated for an additional 6 weeks under the same laboratory conditions, in the absence of insects, to evaluate egg hatching, hole formation, and adult emergence. During the incubation period, newly emerged adults (F_1_ progeny) were checked daily, removed from the Petri dishes immediately upon emergence, and recorded. At the end of six weeks, the number of emergence holes, opened holes, and *A. obtectus* adults emerging from beans were recorded (Figure 1). Representative examples are shown in Figure 2: unhatched (a1, no adult emergence) and hatched holes (a2, adult emergence) and hatched eggs (b1). The beans were reweighed, undamaged beans were counted, and the inhibition rate (IR) and weight loss (WL) of beans were calculated according to the formulas provided by Tegegne [64]. 

Equation (1) was used to calculate the inhibition rate (IR%):

(1) $$IR\left(\%\right)=\frac{\left(Cn-Tn\right)}{Cn}\times 100$$

where IR = Inhibition rate, *C*n = Number of newly emerged insects in the untreated (control) dish; *T*n = Number of newly emerged insects in the treated petri dish.

Equation (2) was used to calculate the weigth loss (WL%):

(2) $$WL\left(\%\right)=\frac{\left(UNd)-(DNu\right)}{U(Nd+Nu)}\times 100$$

where U = weigth of undamaged bean; Nd = number of damaged beans; D = weigth of damaged bean; Nu = number of undamaged beans.

### 2.5. Data Analysis

The obtained mortality data were first corrected for percentage mortality using Abbott’s formula [65].

Equation (3) was used to correct the mortality rates (CMR%):

(3) $$Corrected\;Mortality\;Rates\left(\%\right)=\frac{\left(A-B\right)}{A}\times 100$$

where A = number of live insects in control group %; B = number of live insects in application group %.

All statistical analyses were performed using SPSS software (version 22.0; IBM Corp., Armonk, NY, USA). Results are presented as mean ± standard error (SE). Mortality data were corrected using Abbott’s formula, cumulative mortality rates were calculated, and the data were arcsine square-root transformed prior to statistical analysis. Data in the study (transformed mortality data and bean weight loss, number of eggs per bean, number of holes per bean, number of hatched holes, and number of emerged adults [F_1_ progeny]) were assessed for normality using the Shapiro–Wilk test. Changes in mortality were analyzed using a generalized linear model (GLM) to test the effects of dose, exposure time, and their interaction (dose × time). Differences in bean damage and reproductive indicators among treatments were evaluated using one-way analysis of variance (one-way ANOVA). When significant differences were detected, group means were compared using Tukey’s HSD post hoc test (*p* < 0.05). Median lethal concentrations (LC_50_) and their 95% confidence intervals were estimated by probit regression analysis based on dose–mortality relationships [66,67].

## 3. Results

### 3.1. Mortality and Toxicity of Acanthoscelides obtectus

According to the results of the generalized linear model (GLM) analysis presented in Table 1, adult mortality of *A. obtectus* was significantly affected by the applied dose and exposure time of DE and 1,8-cineole. Both the main effects of dose and time on mortality were statistically significant, and the interaction between dose and time was also found to have a significant effect on mortality (*p* < 0.05).

The LC_50_ values of different doses of DE and 1,8-cineole used in the experiments were calculated [66,67] (Table 2). According to these results, the LC_50_ values of DE and 1,8-cineole at 24 h were determined to be 209.75 ppm and 2.76 ppm, respectively.

### 3.2. Individual and Combined Applications

**Figure 3:**
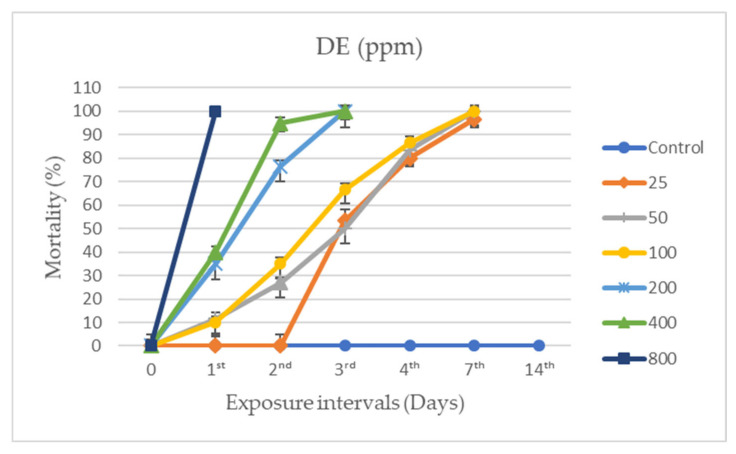
Mean mortality (±SE) of *Acanthoscelides obtectus* exposed to beans treated with individual applications of diatomaceous earth at different doses. DE = diatomaceous earth. Statistical differences were determined at a significance level of *p* < 0.05 using a Tukey HSD post hoc test.

**Figure 4:**
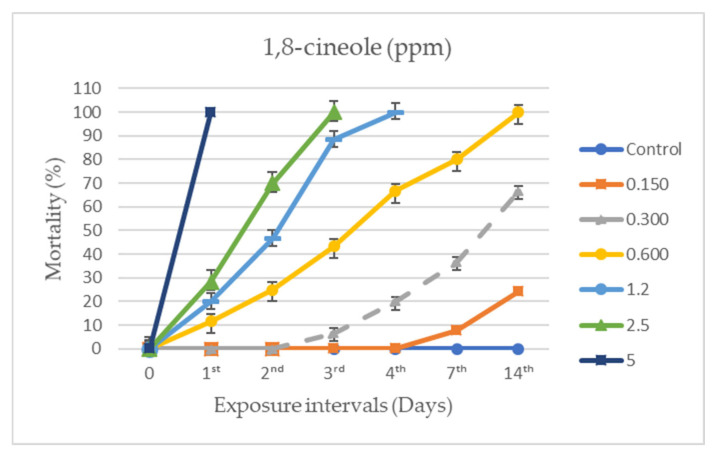
Mean mortality (±SE) of *Acanthoscelides obtectus* exposed to beans treated with individual applications of 1,8-cineole at different doses. Statistical differences were determined at a significance level of *p* < 0.05 using a Tukey HSD post hoc test.

**Figure 5:**
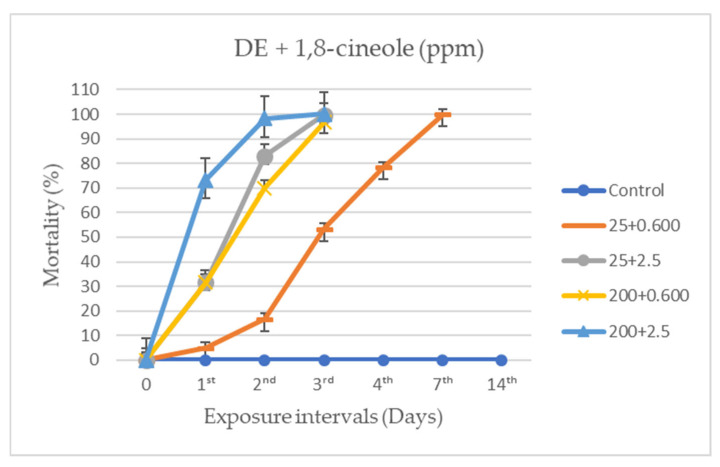
Mean mortality (±SE) of *Acanthoscelides obtectus* exposed to beans treated with different dose combinations of diatomaceous earth and 1,8-cineole. Statistical differences were determined at a significance level of *p* < 0.05 using a Tukey HSD post hoc test. Treatment groups: 25 + 600 = 25 ppm DE + 0.600 ppm 1,8-cineole; 25 + 2.5 = 25 ppm DE + 2.5 ppm 1,8-cineole; 200 + 600 = 200 ppm DE + 0.600 ppm 1,8-cineole; 200 + 2.5 = 200 ppm DE + 2.5 ppm 1,8-cineole.

It was found that both the individual and combined applications of DE and 1,8-cineole exhibited activity against *A. obtectus*, varying according to dose and exposure time (Figure 3, Figure 4 and Figure 5). After 24 h, no mortality was observed in the control groups, whereas 800 ppm DE and 5 ppm 1,8-cineole caused 100% mortality (F_16,85_ = 97.037, *p* < 0.0001).

At 25 ppm DE, the mortality rate was similar to that of the control group during the first 2 days, but increased to 53.33 ± 4.94% by day 3, and reached 96.65 ± 3.33% by day 7 (F_4,25_ = 46.042, *p* < 0.0001). In DE treatments, a pronounced increase in mortality rates was observed particularly after the 200 ppm dose, especially within the first 3 days of exposure. Statistical analysis (GLM) confirmed that dose and exposure time had significant main effects on mortality, while the calculated LC_50_ value for DE (209.75 ppm) revealed a low dose-high effect relationship and supported the time-dependent toxic effect of DE.

In individual applications of 1,8-cineole, mortality rates remained at 24.16 ± 2.10 (F_5,30_ = 58.031, *p*< 0.0001) and 66.49 ± 2.58 (F_5,30_ = 34.268, *p* < 0.0001) at doses of 0.150 and 0.300 ppm, respectively, while mortality within the first 24 h increased significantly with increasing dose (*p* < 0.05). Generalized linear model (GLM) analysis confirmed that both dose and exposure time significantly affected adult mortality, and probit analysis yielded a low LC_50_ value (2.76 ppm). These findings indicate that 1,8-cineole becomes highly effective from 1.2 ppm onward, even after short exposure periods.

In the combined applications of DE and 1,8-cineole, mortality rates increased with exposure time at 25 ppm DE + 0.600 ppm 1,8-cineole and 200 ppm DE + 0.600 ppm 1,8-cineole. Notably, in the treatment with 200 ppm DE + 2.5 ppm 1,8-cineole, the mortality rate reached 73.33 ± 8.81% within the first 24 h, showing a statistically significant difference (F_16,85_ = 97.037, *p* < 0.0001). Nearly 100% of the insect population died within the first 48 h (98.33%, F_14,75_ = 29.416, *p* < 0.0001). Although 100% mortality was achieved on days 7 and 14 at all individual doses except the maximum concentrations, the most pronounced mortality within the first 24 h was observed at 400 ppm DE (40.00 ± 2.58%), 2.5 ppm 1,8-cineole (28.33 ± 4.77%), and, notably, at the combined treatment of 200 ppm DE + 2.5 ppm 1,8-cineole (73.33 ± 8.81%) (F_16,85_= 97.037, *p* < 0.0001).

### 3.3. Weigth Loss

**Figure 6:**
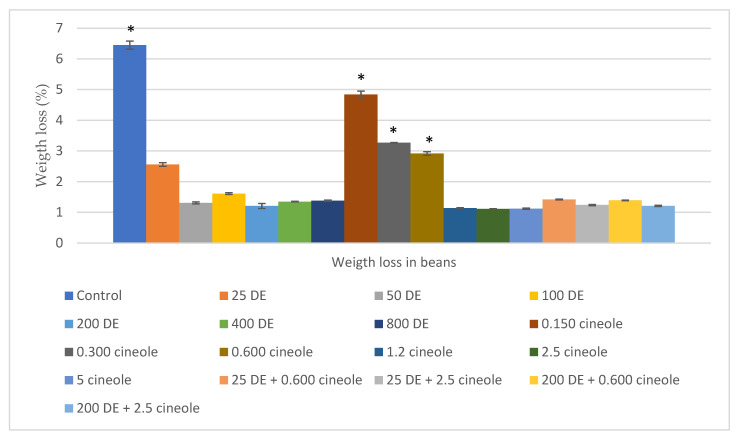
Weight loss of beans after completion of experiments and adult emergence (%). DE: diatomaceous earth. There is a difference between groups marked with an asterisk and groups not marked with an asterisk *. Statistical differences were determined at a significance level of *p* < 0.05 using a Tukey HSD post hoc test [64].

The highest weight loss was observed in the control group, followed by 25 ppm DE, and 0.150, 0.300, and 0.600 ppm 1,8-cineole treatments. Although no adult emergence or hole formation was detected in the DE groups and in the higher dose applications of 1,8-cineole, a slight weight loss was recorded, which was statistically insignificant (*p* > 0.05). This loss is thought to be due to DE-induced moisture reduction in the beans. Bean weight losses were parallel to the number of eggs laid and the number of emerged adults, and the differences were statistically significant (*p* < 0.05) (Figure 6).

### 3.4. F_*1*_ Progeny

In the control group, an average of 5.35 ± 0.65 eggs per bean was recorded, whereas no eggs were observed on the bean or Petri dish surfaces in the DE treatments. After the six-week incubation period, no hole formation or adult emergence was detected in these groups. In the cineole treatments, eggs were observed only at 0.150 ppm (0.92 ± 0.15), 0.300 ppm (0.75 ± 0.26), and 0.600 ppm (0.64 ± 0.50), and these values were significantly different from the control group (*p* < 0.05). In the higher dose cineole applications, no hole formation or adult emergence was observed. In the control group, the mean number of emergence holes was 1.70 ± 0.24 per bean, and the mean F_1_ progeny was 1.40 ± 0.23 per bean. However, in the cineole treatments, the number of emerged adults remained very low and was statistically different from the control (*p* < 0.05) (Table 3). The inhibition rates were found to be above 90% at all doses.

## 4. Discussion

Stored-grain pests cause significant economic losses during grain storage. For many years, insecticides have played a central role in pest control to prevent such losses. However, the development of insecticide resistance in insects, their environmental impacts, and growing concerns about human health necessitate alternative strategies. DE, EOs, their metabolites, and combined applications represent safe and environmentally friendly alternatives to conventional insecticides [68,69,70]. In this study, DE, 1,8-cineole, and DE + 1,8-cineole mixtures were applied to bean grains, and the effects on the mortality rates and F_1_ progeny of *A. obtectus* treated with these beans were determined at different time intervals, while IR and WL were also calculated.

The results revealed that both DE and 1,8-cineole, whether applied individually or in combination, increased the mortality of *A. obtectus* in a dose, and exposure time, dependent manner. In the group treated with the lowest DE dose (25 ppm), mortality was observed on day 3. The insecticidal efficacy of EOs is primarily dependent on the composition and concentration of their major constituents [71]. In the group treated with the lowest 1,8-cineole dose (0.150 ppm), mortality began on day 7. This finding suggests that lower doses have a longer latency period and exert their effects more slowly. Since *A. obtectus* does not feed after adult emergence and is unable to compensate for water loss, even low doses of DE may have resulted in high mortality [72]. At 50, 100, and 200 ppm, mortality was recorded from day 1 onwards. These findings are consistent with previous studies reporting that prolonged exposure of insects to DE increases the contact time with DE particles, thereby enhancing mortality rates [14,73,74,75]. Moreover, this indicates that particularly at lower doses, mortality may require a longer period of exposure before occurring, and that factors such as insect metabolic rate, physiological status, and other environmental conditions should also be taken into account [76].

In contrast to the lower doses, high doses of DE and 1,8-cineole, when applied individually (particularly 800 ppm DE and 5 ppm 1,8-cineole), caused 100% mortality of *A. obtectus* within the first 24 h. The LC_50_ for DE after 24 h was calculated as 209.75 ppm, while the LC_50_ for 1,8-cineole was determined to be 2.76 ppm. At lower doses of DE, 1,8-cineole, and their combined treatments, mortality began from day 1, and the mortality rate increased with longer exposure times. To date, no studies investigating the effects of 1,8-cineole on *A. obtectus* have been reported in the literature; however, research has been conducted on *Callosobruchus maculatus* F. (Coleoptera: Chrysomelidae), which belongs to the same subfamily (Chrysomelidae: Bruchinae). One such study by Et-tazy et al. [77] examined the insecticidal potential of six different essential oils and their major constituents (1,8-cineole, carvacrol, pulegone, and eugenol), as well as their binary mixtures, against *C. maculatus*. In particular, 1,8-cineole (males: LC_50_ = 17.83 μL/L air; females: LC_50_ = 28.08 μL/L air at 48 h) and pulegone (males: LC_50_ = 23.04 μL/L air; females: LC_50_ = 38.25 μL/L air at 48 h) were found to be more potent than the other constituents and essential oils tested. Binary combinations of these compounds, especially 1,8-cineole and carvacrol, caused 70% mortality in males and 60% in females within 48 h, demonstrating greater effectiveness than the individual compounds. These findings are consistent with the results of the present study. In the present study, although low-dose DE + 1,8-cineole applications (e.g., 200 ppm DE + 2.5 ppm 1,8-cineole) did not result in 100% mortality, they produced the highest mortality rate among the treatments at 73.33%, with nearly the entire population dying within 48 h. Alkan et al. [78] investigated the insecticidal effects of two commercial DE products (Turco^®^ and Protector^®^) at different concentrations (100, 200, 400, 600, 800, and 1000 ppm) against *A. obtectus*, and reported 100% mortality after the fourth day at higher doses. In addition, no progeny production was observed at the end of the 55-day incubation period. Jumbo et al. [79] evaluated the effects of DE (0.25, 0.50, 0.75, and 1.00 g/kg^−1^) on the mortality and F_1_ progeny of *A. obtectus* in beans stored at different temperatures (25, 28, 30, 32, and 35 °C). They reported that mortality increased with dose, temperature, and exposure time. Similar to the findings of the present study, their results showed that F_1_ progeny production was reduced by more than 95%. In the present study, it was also observed that both the number of eggs laid and the number of emerged adults were completely suppressed in the DE treatment groups. No eggs were counted, and no adult emergence was recorded at any DE dose. Susurluk and Bütüner [80] investigated the potential control of *Oryzaephilus surinamensis* L. (Coleoptera: Silvanidae) and *A. obtectus* using a local Almina^®^ diatomaceous earth at concentrations of 125, 250, 500, 750, and 1000 ppm. In *O. surinamensis*, the highest mortality (100%) was observed at 1000 ppm after 96 h, whereas in *A. obtectus*, 100% mortality was recorded after 48 h at 500, 750, and 1000 ppm. These findings are partly consistent with the present study and suggest that *A. obtectus* may be more susceptible to desiccation compared to some other insect species, possibly due to the fact that adults do not require feeding after emergence. In the present study, no progeny emergence was observed even at the lowest (25 ppm) and highest (800 ppm) doses of DE when applied alone. Although DE primarily causes insect mortality through desiccation, it is possible that at lower DE doses, the surviving individuals were affected in other ways, such as disruption of courtship behavior, blockage of genital openings, or reduced mating and reproduction [77]. Furthermore, adults emerging from beans do not feed and begin mating within 24 h [72]. Therefore, the presence of DE, due to its silica content, may have accelerated desiccation, hastened mortality in the absence of feeding, and prevented mating opportunities [81].

The number of holes in beans, the number of hatched holes, and the number of emerged adults are important parameters for evaluating the success of treatments [64]. In the present study, no eggs, holes, or emerged adults (per bean) were recorded in any of the DE treatments or in the high-dose applications of 1,8-cineole. Eggs and adults were observed only in the low-dose 1,8-cineole treatments (0.150, 0.300, and 0.600 ppm), but these values were significantly lower compared to the control group and showed statistically significant differences (*p* < 0.05). These findings suggest that the use of DE and 1,8-cineole prevented *A. obtectus* from damaging bean seeds, either by causing excessive water loss and mortality at higher doses or by interfering with mating behavior at lower doses, thereby reducing product loss [82]. In addition, bean weight loss was measured after treatments and was found to be inversely proportional to dose (Figure 6). Although insects in the lower-dose treatments (25, 50, 100 ppm DE and 0.150, 0.300, 0.600 ppm 1,8-cineole) survived up to 7–14 days, they failed to produce F_1_ progeny. This may be attributed to the suppression of courtship and mating behavior, or to the fact that water stress forced the insects to prioritize survival over reproduction. Alternatively, blockage of genital openings may have prevented copulation, or, even if copulation occurred, the insects may not have lived long enough to complete oviposition or egg development [38,39,77]. This phenomenon requires further investigation in future studies.

Monoterpenoids are naturally occurring organic compounds that play a defensive role in plants. They are generally the major constituents of EOs and are known to possess diverse biological activities. Bio-insecticides formulated with EO constituents offer advantages over crude essential oils by providing effective pest control while maintaining environmental safety and sustainability. Monoterpenoids are reported to exert neurotoxic effects on insects, with their ovicidal activity becoming apparent only once the nervous system begins to develop [83]. Changes in the permeability of the vitelline membrane may facilitate the diffusion of EOs into the eggs during embryogenesis, thereby influencing physiological and biochemical processes within the egg. Moreover, beyond their effects during gametogenesis, EOs are also known to interfere with courtship and mating behavior, indirectly reducing oviposition and egg production [36,82,83,84]. In addition, numerous studies have demonstrated the bioactivity of monoterpenoids against major insect pests [85,86,87]. These secondary metabolites generally target the insect nervous system by inhibiting the enzyme acetylcholinesterase. Acetylcholinesterase is essential for neurotransmission, and its inhibition disrupts nerve signal transmission, ultimately leading to insect death [88]. For instance, monoterpenoids such as 1,8-cineole can impair the insect nervous system and cause paralysis [81]. The insecticidal and repellent properties of 1,8-cineole are further supported by previous studies demonstrating its efficacy against a wide range of pests, including *Callosobruchus maculatus*, *Rhyzopertha dominica* F. (Coleoptera: Bostrichidae), *Tribolium castaneum* Herbst (Coleoptera: Tenebrionidae), *Sitophilus oryzae* L. (Coleoptera: Curculionidae), *Spodoptera frugiperda* J.E. Smith (Lepidoptera: Noctuidae), and *Epicauta atomaria* Germar (Coleoptera: Meloidae) [41,57,59,77,89,90]. Moreover, the major constituents of EOs negatively affect insect activities such as feding [51], dispersal [91] and reproduction [52,92,93]. Some compounds can disrupt the digestive system of insects, alter feeding habits, and reduce their attraction to food sources, resulting in decreased feeding and slower development [94,95,96]. Such effects may particularly impair the reproductive cycle and growth rate of pest insects, leading to reduced reproductive performance or various developmental abnormalities [97]. In the present study, 1,8-cineole was also found to exert toxic effects on *A. obtectus*. In particular, 100% mortality was observed by the end of day 1 in the group treated with 5 ppm, by the end of day 3 in the group treated with 2.5 ppm, and by the end of day 4 in the group treated with 1.2 ppm. Tripathi et al. [98] reported that 1,8-cineole exhibited toxic effects on both adults and larvae of *T. castaneum*, significantly reducing egg hatching, larval survival, and adult emergence. Similarly, Obeng-Ofori et al. [54] demonstrated that 1,8-cineole was effective as a repellent, toxicant, and grain protectant against *Sitophilus granarius* L. (Coleoptera: Curculionidae), *S. zeamais* Motschulsky (Coleoptera: Curculionidae), *T. castaneum*, and *Prostephanus truncatus* Horn (Coleoptera: Bostrichidae). In their study, egg development, immature stages, and progeny emergence were completely inhibited in treated grains. These findings are in line with the results of the present study with respect to mortality rates, oviposition, and F_1_ progeny production.

EOs and their metabolites, monoterpenes, have attracted increasing attention as low-toxicity, environmentally friendly alternatives to synthetic repellents and insecticides against insect pests. However, their practical application in stored grain protection remains limited due to their relatively low efficacy and interference with the organoleptic properties of grains. At the same time, it has been reported that high doses of DE are required to achieve satisfactory levels of protection against insect pests [41,59]. Nevertheless DE concentrations of 1000 ppm and above have been shown to reduce the hectoliter weight of grains and are therefore not recommended [73]. Consequently, recent research has increasingly focused on combination strategies involving agents such as EOs, inert dusts, and fungi. In the present study, the reduced-dose combinations also appear to hold potential for protecting stored products against insect pests. Both DE and 1,8-cineole were found to have potent insecticidal activity against *A. obtectus*, particularly at high concentrations. Furthermore, in combined treatments, a mixture of 200 ppm DE + 2.5 ppm 1,8-cineole resulted in the highest mortality. These findings suggest that combined DE and 1,8-cineole application exhibits greater insecticidal activity, with higher doses being more effective. The strong insecticidal activity may be attributed to the distinct mechanisms of action of these two agents: while DE induces desiccation and mortality through excessive water loss, 1,8-cineole primarily affects the insect nervous system, leading to rapid death. The enhanced efficacy of the combinations compared with the individual treatments further supports this synergism. For example, although the combination of 25 ppm DE + 0.600 ppm 1,8-cineole caused mortality from the first day (5.00 ± 2.23), complete mortality was achieved only by day 7. In contrast, the combination of 200 ppm DE + 2.5 ppm 1,8-cineole resulted in a mortality rate of 73.33 ± 8.81% at the end of the first day. The increased effectiveness of the combinations can be explained by the complementary mechanisms of action: DE provides a physical barrier leading to dehydration, whereas 1,8-cineole exerts both contact toxicity and fumigant action via the vapor phase, thereby disrupting the insect’s respiratory and nervous systems. This dual interaction accelerates and enhances mortality in *A. obtectus* [76].

As demonstrated in similar studies [58,99], the combined application of the main constituents of EOs or their use in conjunction with inert dusts has emerged as a more powerful strategy for pest control. For instance, in a study investigating the combined effects of two commercial DE-based insecticides, Protect-It^TM^ and SilicoSec^®^, containing eugenol and cinnamaldehyde, against *C. maculatus* and *S. oryzae*, the combination of Protect-It™ and cinnamaldehyde did not exhibit a synergistic effect against *C. maculatus*. In contrast, all combinations showed synergistic effects against *S. oryzae* [42]. Similarly, Yang et al. [100] demonstrated that the simultaneous application of garlic essential oil and DE possesses significant potential in controlling *S. oryzae* and *T. castaneum*. Moreover, a natural SilicoSec^®^ formulation enriched with plant-derived components, known as Form N, was reported to cause higher mortality rates in *S. oryzae*, *R. dominica*, and *T. castaneum*, particularly in barley seeds, compared to DE alone [101]. The differences in the activity of monoterpenes and DE among various insect species may be due to the ability of these products to penetrate the insect body, the insect’s capacity to metabolize such compounds, and the variation in their physiological responses [25].

In a study evaluating the insecticidal efficacy of *Trichoderma harzianum* Rifai (0.0, 3.3 × 10^6^, 6.6 × 10^6^, and 2.1 × 10^7^ spores/kg) and DE (0, 200, 400, and 800 ppm) against *A. obtectus*, the highest mortality rate (93.88%) was obtained seven days after treatment with 800 ppm DE and 2.1 × 10^7^ spores/kg of *T. harzianum*, and the emergence of the F_1_ progeny was found to be suppressed [102]. In the present study, 100% mortality was observed within the first 24 h in groups treated with 800 ppm DE. In the study of Gad et al. [102], nearly 100% mortality was achieved only on the 7th day in the group treated with 800 ppm DE, which may be attributed either to the combined use of an additional agent or to differences in DE particle size (the SiO_2_ content was 80.6% in the present study compared with 46.37% in their study. Indeed, smaller DE particles are known to adhere more effectively to the insect cuticle, increasing abrasion and enhancing water loss, thereby improving insecticidal performance [75]. These findings highlight that the physicochemical properties of DE, particularly particle size and silica content, play a crucial role in determining its efficacy and must be carefully considered in pest management strategies. Adarkwah et al. [25] investigated the toxicity of *Eugenia aromatica* and *Moringa oleifera* powders, either alone or in combination with an enhanced diatomaceous earth formulation (Probe-A^®^ DE, containing 89.0% SiO_2_ and 5% silica aerogel), against adults of *S. granarius*, *T. castaneum* and *A. obtectus*. They reported that adult mortality was observed up to 7 days, with *A. obtectus* being the most susceptible species to the plant powders. Furthermore, the combined mixture of plant powders and DE provided faster control of insects compared to plant powders alone and also caused a significant reduction in F_1_ progeny emergence. In the present study, *A. obtectus* was likewise found to be more sensitive to DE than to 1,8-cineole, even at relatively low doses. Mortality in adults exposed to 25 ppm DE began as early as the 3rd day, whereas at the lowest 1,8-cineole dose (0.150 ppm), mortality was not detected until after the 7th day. The mortality rate on day 14 was determined to be only 24.16 ± 2.10. Although DE alone appeared to be effective at controlling *A. obtectus*, the combination treatments were comparatively more advantageous. However, maximum doses of DE are often not recommended due to issues such as product residues and corrosive effects, while high doses of monoterpenes may cause irritant effects in humans [103,104,105]. Importantly, in the present study, lower-dose combinations (particularly 200 ppm DE + 0.600 ppm 1,8-cineole and 200 ppm DE + 2.5 ppm 1,8-cineole) produced effects comparable to those obtained with maximum single treatments. The fact that such reduced-dose DE + 1,8-cineole combinations yielded mortality levels similar to those achieved with maximum doses when applied alone highlights their potential as promising agents for the control of *A. obtectus*.

## 5. Conclusions

The findings of this study demonstrate that diatomaceous earth (DE) and 1,8-cineole are effective against *A. obtectus*, and that their combined applications notably increase adult mortality and significantly suppress F_1_ progeny emergence. Both DE and 1,8-cineole have long been recognized as promising protectants against a wide range of stored-product insect pests. Despite their advantages in terms of environmental compatibility and reduced risks to human health, these agents may cause adverse effects on treated commodities when applied at high doses. To mitigate these limitations while maintaining efficacy, the present study explores the use of reduced doses of DE and 1,8-cineole for the control of *A. obtectus*. The results indicate that both agents are individually effective and that their combinations produce pronounced insecticidal effects, resulting in high mortality rates and substantial suppression of F_1_ progeny. These findings highlight the potential of environmentally friendly and ecologically sound alternatives for the management of insect pests in stored agricultural products such as beans. The outcomes are also consistent with the principles of sustainable agriculture, particularly by addressing challenges associated with the high volatility and low water solubility of essential oils, as well as the application constraints of DE. Nevertheless, it should be emphasized that these results were obtained under controlled laboratory conditions and should be interpreted with caution before extrapolation to field or commercial storage environments. Although the combined use of DE and 1,8-cineole at reduced doses appears to enhance efficacy while potentially lowering adverse effects, critical aspects such as practical applicability under real storage conditions, possible impacts on product quality, residue levels, and food safety have not yet been fully evaluated. In addition, the high volatility of essential oils and the physical characteristics of DE may pose technical and economic challenges for large-scale applications. Therefore, while the present findings suggest that DE and 1,8-cineole combinations represent a promising alternative strategy for the management of stored-product pests, further validation through long-term storage experiments, semi-field, and field trials is required prior to practical implementation. Future studies should focus on optimizing dose combinations, refining application methods, assessing scalability, and evaluating food safety and residue dynamics to support the development of sustainable and effective pest management strategies.

## Figures and Tables

**Table 1:** Results of factorial analysis (GLM) showing the main effects and interactions of dose and exposure time on mortality of adult *Acanthoscelides obtectus*.

Source	Degree of Freedom	F-Value	*p*-Value
Dose	16	36.886	<0.001
Time	5	233.583	<0.001
Dose × Time	80	49.287	<0.001
Total	612		

**Table 2:** Toxicological parameters of *Acanthoscelides obtectus* exposed to diatomaceous earth and 1,8-cineole.

Treatment	Days	LC_50_ (ppm) (95% Fiducial Limit)	Slope ± S.E.	*p* Value	Intercept
DE	1st	209.75 (127.31–343.94)	1.645 ± 0.110	0.025	1.182
	2nd	109.81 (73.77–163.46)	2.277 ± 0.088	0.114	0.354
	3rd	83.85 (50.90–138.13)	−1.610 ± 0.111	<0.001	8.097
	4th	24.95 (5.15–120.92)	−0.474 ± 0.350	<0.001	5.662
	7th	--	--	--	--
	14th	--	--	--	--
1,8-cineole	1st	2.76 (1.46–5.22)	1.320 ± 0.141	<0.001	4.416
	2nd	1.44 (0.94–2.21)	2.113 ± 0.094	0.017	4.661
	3rd	1.42 (0.84–2.41)	1.516 ± 0.117	0.010	4.767
	4th	0.00009 (0.0000–0.01128)	−0.161 ± 1.072	<0.001	4.347
	7th	6.30 (1.83–21.60)	0.632 ± 0.273	<0.001	4.495
	14th	0.0091 (0.00061–0.13495)	−0.283 ± 0.597	0.001	4.423

LC_50_: Concentration of a chemical in inhaled air that causes 50% mortality in a group of test organisms under specified conditions. DE: Diatomaceous earth. --: Data not calculable. S.E.: Standard Error. At later observation days, high mortality levels resulted in reduced dose–response gradients, leading to negative slope values and wide confidence intervals. Median lethal concentrations (LC_50_) and their 95% confidence intervals were estimated by probit regression analysis based on dose–mortality relationships.

**Table 3:** Effects of diatomaceous earth and of 1,8-cineole on oviposition, hole formation, F_1_ progeny and inhibition rate (IR%) of *Acanthoscelides obtectus*.

Treatments	Doses (ppm)	Number of Eggs	Number of Unhatced Holes	Number of Hatched Holes	F_1_ Progeny	Inhibition Rate (IR%)
			Mean ± S. E.			
DE	Control	5.35 ± 0.65 ^A^	2.54 ± 0.21 ^A^	1.70 ± 0.24 ^A^	1.40 ± 0.23 ^A^	-
	25	-	-	-	-	100.00
	50	-	-	-	-	100.00
	100	-	-	-	-	100.00
	200	-	-	-	-	100.00
	400	-	-	-	-	100.00
	800	-	-	-	-	100.00
1,8-cineole	0.150	0.92 ± 0,15 ^B^	1.35 ± 0.11 ^B^	0.67 ± 0.06 ^B^	0.09 ± 0.02 ^B^	93.50
	0.300	0.75 ± 0.26 ^B^	0.53 ± 0.09 ^C^	0.31 ± 0.05 ^B^	0.06 ± 0.01 ^B^	95.27
	0.600	0.64 ± 0.50 ^B^	0.15 ± 0.02 ^C^	0.15 ± 0.05 ^B^	0.03 ± 0.01 ^B^	97.65
	1.2	-	-	-	-	100.00
	2.5	-	-	-	-	100.00
	5	-	-	-	-	100.00
DE + 1,8-cineole	25 + 0.600	-	-	-	-	100.00
	25 + 2.5	-	-	-	-	100.00
	200 + 0.600	-	-	-	-	100.00
	200 + 2.5	-	-	-	-	100.00
	F_3,20_ =	42.716	65.013	24.901	31.460	
	*p*	<0.001	<0.001	<0.001	<0.001	

There is no significant difference at the *p* < 0.05 level between means shown with the same capital letter in the same column. Statistical differences were determined at a significance level of *p* < 0.05 using a Tukey HSD post hoc test. S.E.: Standard Error.

## Data Availability

The original contributions presented in this study are included in the article. Further inquiries can be directed to the corresponding author.
